# Primary Ventral Hernia as an Uncommon Cause of Small Bowel Obstruction in a Surgically Naïve Patient: A Clinical Case Report

**DOI:** 10.7759/cureus.100419

**Published:** 2025-12-30

**Authors:** John Salib, Mark Salib, Frederick Tiesenga

**Affiliations:** 1 School of Medicine, St. George's University School of Medicine, St. George's, GRD; 2 General Surgery, West Suburban Medical Center, Chicago, USA

**Keywords:** abdominal wall hernia, atypical patient presentation, early recognition and intervention, emergency general surgery, hernia incarceration, incarcerated ventral hernia, open hernia repair, operative management, small bowel obstruction, surgical decision-making

## Abstract

Small bowel obstruction (SBO) is a common surgical emergency with multiple etiologies, including adhesions, malignancy, and hernias. In patients with no prior abdominal surgery, incarcerated hernias are an uncommon but important cause of mechanical obstruction. We report the case of a 44-year-old African-American woman with no surgical history who presented with progressive abdominal pain, bilious vomiting, and a nonreducible ventral abdominal wall mass. Prompt surgical exploration revealed a 4-cm incarcerated ventral hernia with compromised omentum. Early recognition and timely operative intervention prevented bowel ischemia and resulted in an uncomplicated recovery. This case highlights the importance of considering ventral hernia as a cause of SBO in patients with a surgically naïve abdomen and reinforces the need for rapid operative management to prevent serious complications.

## Introduction

Ventral hernias, which include primary umbilical and epigastric defects as well as incisional hernias, represent a significant subset of abdominal wall hernias and contribute greatly to acute abdominal presentations [1]. They account for a substantial proportion of cases that progress to surgical emergencies. Their clinical importance lies in the risk of incarceration, in which herniated bowel becomes trapped within a rigid fascial defect and loses venous and lymphatic outflow. Continued compression can progress to arterial compromise, strangulation, and eventual bowel necrosis if left untreated [1,2]. Despite advances in elective hernia repair techniques, ventral hernias continue to generate a high burden of emergency surgical cases due to these complications.

Small bowel obstructions (SBOs) remain one of the most common causes of emergency general surgery admissions [3,4]. Although postoperative adhesions are the leading etiology overall, incarcerated hernias are the most frequent cause in patients without a history of abdominal surgery. This form of obstruction is hazardous because bowel compromise can occur more rapidly and unpredictably than in adhesive disease. Mortality increases significantly when intervention is delayed beyond the early window of ischemic progression, which highlights the importance of prompt recognition and surgical management [3,5].

Diagnosis can be difficult when ventral hernias present atypically. Unlike inguinal hernias, which often produce a distinct bulge, ventral hernias may be small, deep, or obscured by adipose tissue, especially in women and individuals with higher body mass index (BMI) [1,6]. These subtle hernias may mimic benign soft tissue masses or minor contour irregularities and can often be easily overlooked during a brief physical assessment. Their presenting symptoms, including intermittent abdominal discomfort, bloating, nausea, or brief episodes of obstruction, are often nonspecific and overlap with gastrointestinal, hepatobiliary, or gynecologic conditions [5,7]. In women, diagnostic delays are even more common because such symptoms may be initially attributed to ovarian pathology, urinary tract disorders, or functional gastrointestinal syndromes [4].

Imaging plays a critical role in establishing the diagnosis. However, even computed tomography (CT) can miss minor or partially reducible defects if the study is not closely evaluated for focal fascial discontinuity, bowel angulation, or small volumes of entrapped omentum [8,9]. For patients without prior abdominal surgery, clinicians must maintain an exceptionally high index of suspicion for hernia-related obstruction, given the limited number of alternative mechanical causes.

We present the case of a 44-year-old African American woman with no previous surgical history who developed an acute SBO secondary to an incarcerated ventral hernia. This case highlights the importance of considering ventral hernia-related obstruction, even in patients who do not fit traditional demographic patterns and may lack a clearly identifiable abdominal wall defect.

## Case presentation

A 44-year-old African American woman presented to the emergency department with a 24-hour history of progressively worsening abdominal pain, persistent nausea, multiple episodes of bilious emesis, and complete absence of flatus or bowel movements. She described the pain as diffuse, crampy, and increasingly severe since onset. Her past medical and surgical histories were unremarkable, and she reported no chronic illnesses or prior abdominal operations. The only noted allergy was to ACE inhibitors, which previously caused angioedema. She denied chest pain, dyspnea, hematochezia, or urinary symptoms.

On arrival, the patient appeared acutely ill and visibly uncomfortable. Vital signs on presentation were notable for a temperature of 38.3 °C, heart rate of 112 beats per minute, blood pressure of 128/76 mmHg, respiratory rate of 20 breaths per minute, and oxygen saturation of 98% on room air, raising concern for an early systemic inflammatory response. Abdominal examination revealed a distended abdomen with diffuse tenderness to both superficial and deep palpation, accompanied by voluntary guarding and mild rebound tenderness, suggesting evolving peritonitis. A firm, tender, nonreducible mass was palpated in the right lower abdominal wall without overlying skin changes. Given the combination of systemic instability, signs of peritoneal irritation, and the presence of a nonreducible ventral hernia, the clinical picture was highly concerning for a strangulated hernia with compromised bowel.

Laboratory evaluation on admission (Table 1) revealed a leukocytosis of 14,900/µL with marked neutrophil predominance (82%), consistent with an acute inflammatory or infectious process. Hemoglobin, hematocrit, and platelet counts were within normal limits. Serum electrolytes were unremarkable, and both blood urea nitrogen and creatinine levels were within normal ranges, indicating preserved renal function despite ongoing vomiting. Liver enzymes and bilirubin were also normal. A mildly elevated serum lactate of 2.4 mmol/L raised concern for early tissue hypoperfusion or evolving bowel compromise in the setting of suspected obstruction. A urine pregnancy test was negative, and urinalysis was largely unremarkable aside from trace ketones, likely reflecting reduced oral intake.

**Table 1:** Laboratory results on admission. Admission laboratory values for a patient presenting with an incarcerated ventral hernia and small bowel obstruction. Findings include leukocytosis with neutrophil predominance, consistent with an acute inflammatory response; electrolyte and BUN abnormalities suggestive of dehydration secondary to vomiting; and a mildly elevated lactate level, raising concern for early tissue hypoperfusion in the setting of possible bowel compromise. CMP, comprehensive metabolic panel; Hgb dipstick, hemoglobin dipstick; CBC, complete blood count

Lab test	Analyte (Abbreviation)	Result	Reference range
Complete blood count (CBC)	White blood cell count (WBC)	14,900/µL	4,000-11,000/µL
	Hemoglobin (Hgb)	13.2 g/dL	12.0-16.0 g/dL
	Hematocrit (Hct)	39%	36%-46%
	Platelets (Plt)	260 × 10³/µL	150-400 × 10³/µL
CBC differential	Neutrophils (Neut)	82%	40%-70%
	Lymphocytes (Lymph)	12%	20%-40%
	Monocytes (Mono)	5%	2%-8%
	Eosinophils (Eos)	1%	1%-4%
	Basophils (Baso)	0%	0-1%
Comprehensive metabolic panel (CMP)	Sodium (Na⁺)	137 mmol/L	135-145 mmol/L
	Potassium (K⁺)	3.9 mmol/L	3.5-5.0 mmol/L
	Chloride (Cl⁻)	102 mmol/L	98-107 mmol/L
	Bicarbonate (HCO₃⁻)	23 mmol/L	22-29 mmol/L
	Blood urea nitrogen (BUN)	12 mg/dL	7-20 mg/dL
	Creatinine (Cr)	0.8 mg/dL	0.6-1.3 mg/dL
	Glucose (Glu)	108 mg/dL	70–140 mg/dL
	Calcium (Ca²⁺)	9.1 mg/dL	8.5-10.5 mg/dL
	Aspartate aminotransferase (AST)	22 U/L	10-40 U/L
	Alanine aminotransferase (ALT)	18 U/L	7-56 U/L
	Alkaline phosphatase (ALP)	78 U/L	40-130 U/L
	Total bilirubin (Tbili)	0.8 mg/dL	0.2-1.2 mg/dL
Urinalysis (UA)	Appearance/Color	Yellow, clear	Clear
	Specific gravity (SG)	1.02	1.005-1.030
	pH	6	4.5-8.0
	Leukocyte Esterase (LE)	Negative	Negative
	Nitrites (Nit)	Negative	Negative
	Protein (Prot)	Negative	Negative
	Glucose (Glu)	Negative	Negative
	Ketones (Keto)	Trace	Negative
	Blood (Hgb dipstick)	Negative	Negative
Pregnancy test	Urine pregnancy test (UPT)	Negative	Negative
Other relevant labs	Lactate (Lac)	2.4 mmol/L	0.5-2.0 mmol/L

Given the evolving peritonitic manifestations, leukocytosis with neutrophil predominance, and rising concern for bowel compromise, the clinical picture was consistent with an acute surgical abdomen. To avoid delaying definitive intervention, additional imaging was deferred, and the patient was taken emergently to the operating room for exploratory surgery and definitive management.

**Figure 1:**
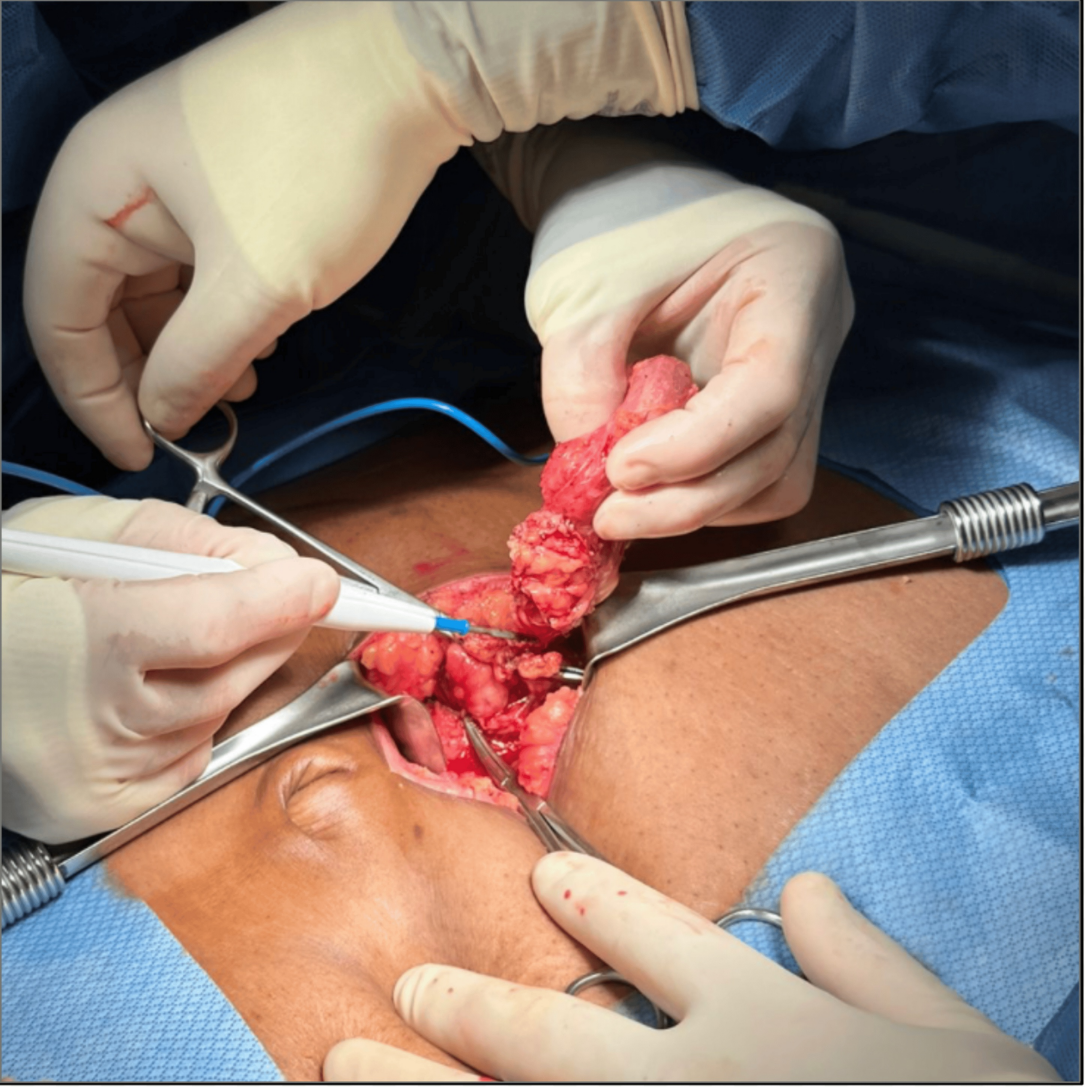
Intraoperative exposure of incarcerated omentum within a ventral hernia defect. This photograph demonstrates the surgical exploration of a 4-cm ventral hernia containing tightly incarcerated omentum. After skin and fascial incision, the hernia sac was opened to reveal congested, edematous omental tissue firmly trapped within the defect.

**Figure 2:**
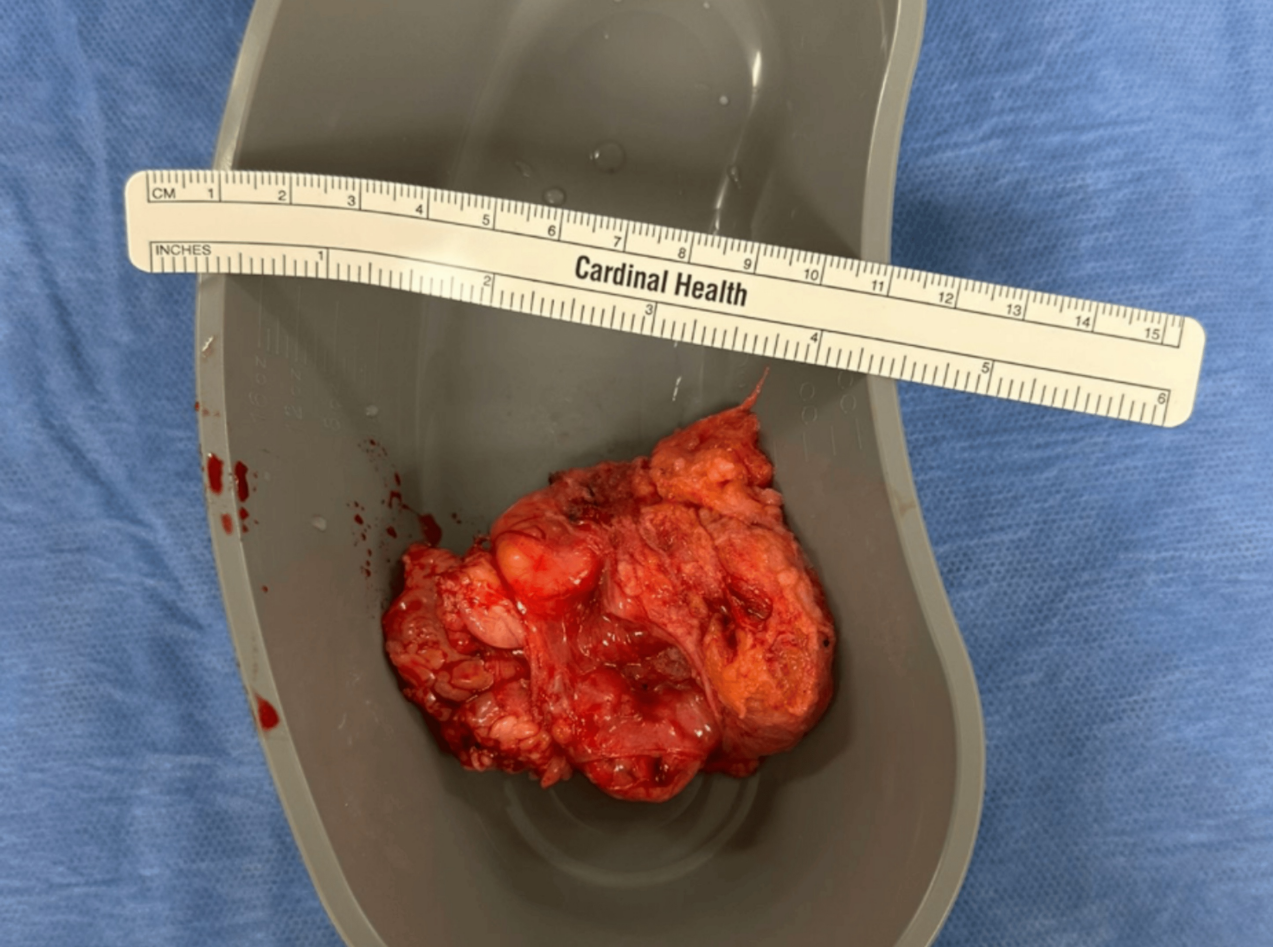
Resected incarcerated ventral hernia sac and omentum. Gross specimen showing the excised ventral hernia sac along with the portion of incarcerated omentum that was unreducible. The remainder of the omentum and abdominal contents were returned to the peritoneal cavity.

Upon entering the abdominal cavity, a 4-cm ventral fascial defect was recognized, incorporating a tightly incarcerated omentum with evidence of venous congestion and early ischemic changes. The involved omental segment was firmly adherent within the hernia sac, preventing reduction of the hernia. Careful dissection was accomplished to free the ensnared tissue, followed by reduction of the hernia contents (Figure 1). The nonviable portion of the omentum was resected (Figure 2), and the specimen was sent for pathologic evaluation. Given the emergent setting, the presence of ischemic and necrotic omental tissue, and the increased risk of mesh infection in a potentially contaminated field, the decision was made to perform primary fascial closure using non-absorbable sutures. Although mesh reinforcement is generally recommended for ventral hernia defects larger than 2 cm to reduce recurrence risk, this approach was deferred in favor of damage-control repair, with consideration for elective definitive reconstruction if clinically indicated. The patient tolerated the procedure without complications.

**Figure 3:**
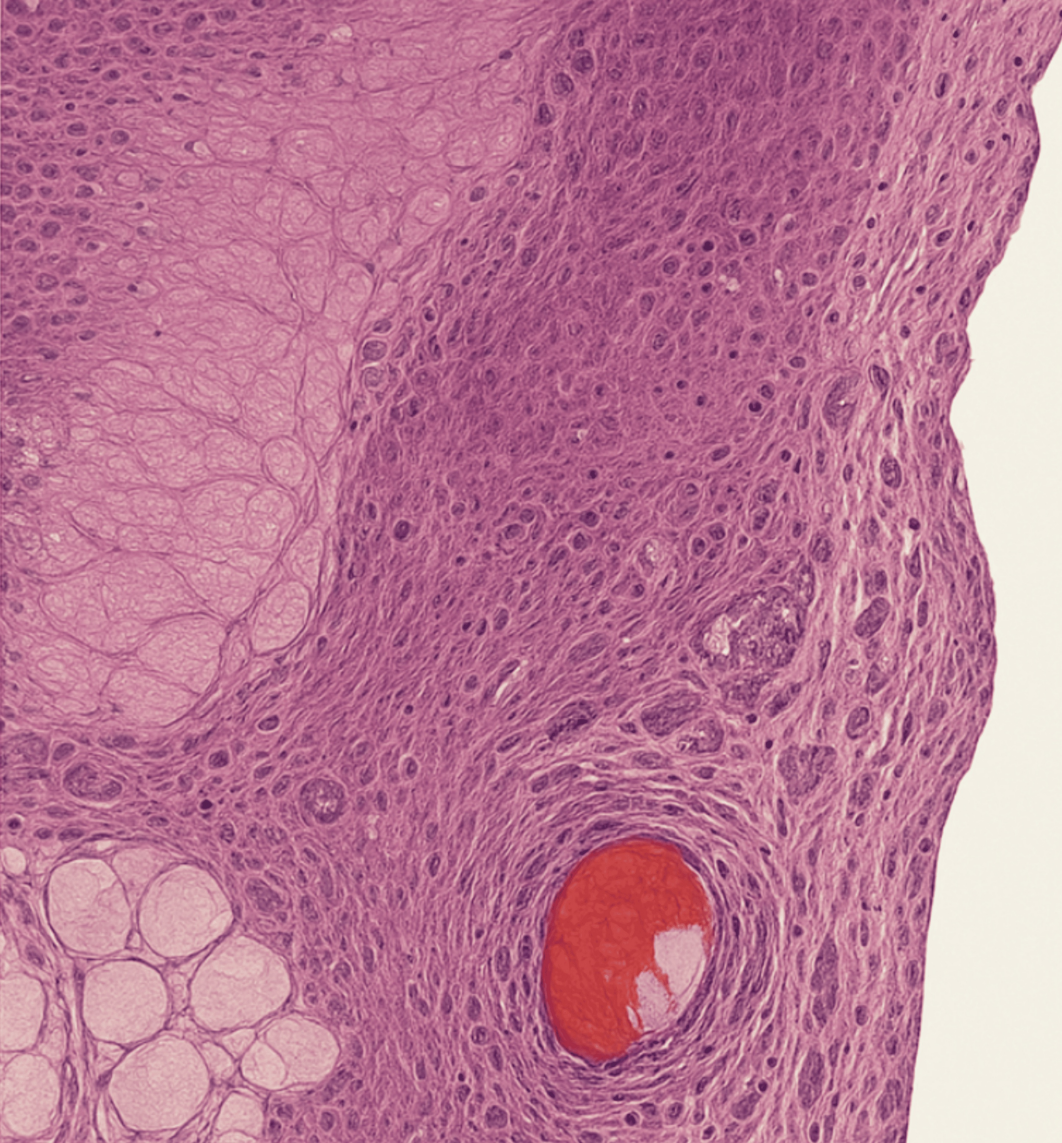
Hematoxylin and eosin (H&E)-stained photomicrograph of the hernia sac wall with associated fibroadipose tissue (×100 magnification). Hematoxylin and eosin (H&E)-stained photomicrograph demonstrating the histologic architecture of the hernia sac wall. The image shows dense fibrocollagenous tissue with interspersed adipose lobules. Areas of vascular congestion, focal hemorrhage, and dilated blood vessels are present, findings consistent with ischemic changes secondary to incarceration. No evidence of malignancy, necrotizing infection, or alternative pathologic processes is identified.

Pathologic examination of the resected omental specimen demonstrated benign fibroadipose tissue with focal areas of congestion and hemorrhage, consistent with ischemic changes secondary to incarceration (Figure 3). No evidence of malignancy, necrotizing infection, or other pathologic processes was identified.

The patient tolerated the procedure well without complications. Her postoperative course was uncomplicated, and she was discharged on postoperative day 3 after achieving adequate pain control, tolerating oral intake, and demonstrating stable vital signs and return of bowel function. At her two-week outpatient follow-up, she reported no new concerns, her incision was healing appropriately, and she had fully resumed normal daily activities without recurrence of symptoms.

## Discussion

Ventral hernias may be broadly categorized into primary ventral hernias, which include umbilical, epigastric, and Spigelian defects, and incisional hernias, which arise at the site of prior abdominal surgery. Primary ventral hernias result from congenital or acquired weaknesses in the abdominal wall fascia. Commonly associated factors include increased intra-abdominal pressure, connective tissue disorders, obesity, and pregnancy [5-8]. In contrast, incisional hernias reflect failure of fascial healing following surgery and account for the majority of ventral hernia repairs in adults [8,9]. Consequently, much of the existing literature on ventral hernia complications, including incarceration and obstruction, is disproportionately derived from incisional hernia cohorts.

Although primary ventral hernias are common in adults, particularly umbilical defects, progression to acute incarceration causing obstructive symptoms is comparatively uncommon [8]. Many primary defects remain asymptomatic or present with vague, intermittent discomfort and are often managed electively [10-12]. Incarceration risk appears to be influenced by defect size, with smaller defects (approximately 2-4 cm) carrying a higher risk due to their tendency to entrap herniated contents, while preventing spontaneous reduction [6-9]. Primary ventral hernias are less likely to involve adhesions or complex intra-abdominal anatomy, making acute obstruction less expected in surgically naïve patients.

The present case represents an uncommon clinical scenario in which a primary ventral hernia in a surgically naïve adult progressed to acute incarceration, causing obstructive symptoms. The absence of prior abdominal surgery, a known history of hernia, or traditional predisposing factors reduced initial clinical suspicion and increased the risk of diagnostic delay. Furthermore, the relatively small defect size (4 cm) likely contributed to the entrapment of omentum and subsequent obstruction. This case highlights the importance of distinguishing between primary and incisional ventral hernias when evaluating patients with acute abdominal symptoms, as reliance on incisional hernia-dominant data may underestimate the potential severity of primary defects in certain patients.

Several differential diagnoses must be considered when evaluating acute abdominal pain and suspected obstruction, particularly in patients without prior abdominal surgery (Table 2). Adhesive disease remains the most common overall cause of obstruction; however, it is far less likely in patients who have never undergone abdominal operations, making it improbable in this case [4,5,7]. Internal hernias may present with acute obstruction but are typically associated with prior bariatric or colorectal procedures and are uncommon in surgically naïve patients [6,8]. Gastrointestinal malignancy can also cause obstruction, but tumors generally produce a more gradual, progressive onset of symptoms rather than sudden, severe abdominal pain. Volvulus is another consideration; however, it often presents with characteristic clinical signs, such as acute onset with intermittent pain and abdominal distension, and is rare in patients without predisposing anatomical abnormalities [9]. In this patient, surgical exploration revealed a focal anterior abdominal wall defect containing incarcerated omentum, which provided the definitive source of obstruction. This highlights the importance of including incarcerated ventral hernias in the differential diagnosis for obstructive symptoms, even in patients who lack classic risk factors or prior surgical history.

**Table 2:** Differential diagnoses for acute obstructive abdominal symptoms in a surgically naïve patient. This table summarizes the main differential diagnoses considered when evaluating a patient presenting with acute obstructive abdominal symptoms. For each condition, predisposing factors, typical symptom onset, key clinical findings, and reasons for lower likelihood in this patient are listed. Definitive diagnosis was confirmed intraoperatively by identifying a primary ventral hernia with incarcerated omentum [4-10].

Condition	Typical clinical setting/predisposing factors	Symptom onset and key features	Clinical findings	Why less likely in this patient
Adhesive small bowel obstruction	Prior abdominal or pelvic surgery, adhesions from inflammation, or prior operations	Gradual or intermittent crampy abdominal pain, bloating, nausea, vomiting	Distended abdomen, high-pitched bowel sounds, tenderness	No prior surgical history; adhesions are highly improbable
Internal hernia	Post-bariatric surgery, colorectal surgery, and congenital mesenteric defects	Acute or intermittent obstruction may mimic adhesive SBO	Intermittent pain, vomiting, possible palpable mass if protrusion occurs	No history of prior abdominal surgery or anatomic abnormalities; unlikely in a surgically naïve patient
Malignant bowel obstruction	History of gastrointestinal or intra-abdominal malignancy, age >50	Progressive, subacute symptoms: weight loss, early satiety	Palpable mass, anemia, subtle abdominal distension	Patient is younger, no known malignancy, symptoms were sudden and severe rather than progressive
Volvulus	Chronic constipation, elongated mesentery, prior volvulus, redundant colon	Acute colicky abdominal pain, nausea, vomiting, and distension	Intermittent severe pain, tympanic abdomen, and possible signs of peritonitis	No predisposing anatomy or history; presentation inconsistent with typical volvulus
Incarcerated primary ventral hernia (diagnosis)	Can occur with or without classic risk factors; defect 3-4 cm at higher risk; obesity, female sex, and increased intra-abdominal pressure.	Acute, severe abdominal pain; nausea, vomiting, obstipation	Palpable anterior abdominal wall defect, localized tenderness, signs of obstruction	Surgical exploration confirmed a 4-cm ventral hernia containing incarcerated omentum; diagnosis established intraoperatively

Pathophysiologically, incarceration occurs when intra-abdominal contents become trapped within a hernia defect and cannot be reduced, leading to venous congestion, rising compartmental pressure, and potentially arterial compromise, strangulation, and necrosis if untreated [8]. Defect size is a key determinant of risk. Defects measuring between 3 and 4 cm are particularly prone to incarceration because they are sufficiently large to allow herniation yet small enough to entrap omentum [10]. Adhesions within the hernia sac may further fixate herniated tissue, increasing the likelihood of vascular compromise [10,11].

These clinical mechanisms are supported by data suggesting that defects between 3 and 4 cm carry nearly a threefold increase in the risk of incarceration compared to defects that are either smaller or larger [11]. The same study identified additional contributors, including female sex and elevated body mass index, which amplified risk, particularly in incisional hernias. These findings highlight the interplay between anatomical and patient-specific factors, reinforcing the importance of individualized assessment when evaluating hernia-related complications [12].

A nationwide cohort study of more than 30,000 patients managed nonoperatively reported an incarceration incidence of 2.3 %, confirming that, although relatively uncommon, incarceration remains a clinically meaningful risk [12]. Independent predictors included age over 40, female sex, African American race, and obesity. These demographic variables should be incorporated into risk stratification. Together, these studies emphasize that patients without a classic surgical history may still be at elevated risk and warrant careful evaluation [13].

Our patient lacked most conventional risk factors yet developed an incarcerated hernia causing obstructive symptoms, demonstrating that atypical presentations should not exclude hernias from the differential diagnosis. Early identification is critical because incarcerated hernias remain a leading cause of acute obstruction, and delays increase the likelihood of strangulation, perforation, and sepsis [14-16]. While imaging can aid diagnosis in less clear cases, prompt surgical evaluation remains essential when there is a clear acute surgical abdomen.

In this case, prompt open repair with partial omentectomy revealed nonviable omentum but viable bowel, allowing an uncomplicated recovery. This case highlights the importance of thorough assessment and careful differential consideration, especially in patients who fall outside established risk profiles.

## Conclusions

This case demonstrates an atypical presentation of SBO caused by a primary ventral hernia in a surgically naïve adult. This scenario is uncommon, given that most SBOs in adults are due to postoperative adhesions or other more typical causes. The patient lacked conventional risk factors such as prior abdominal surgery, obesity, or a known hernia, and the defect was relatively small. However, it still led to acute incarceration of the omentum and progression to obstruction. This underscores the clinical importance of including primary ventral hernias in the differential diagnosis of SBO, even in patients who do not fit classic risk profiles. Imaging was deferred due to clear signs of an acute surgical abdomen, and prompt operative intervention allowed for successful management without delay. This case highlights the need for high clinical vigilance, careful physical assessment, and individualized decision-making in the evaluation and management of SBO, particularly in surgically naïve patients, and reinforces that rare but significant presentations can have serious consequences if not recognized promptly.
